# Supplementary material for: Electricity generation from carbon dioxide adsorption by spatially nanoconfined ion separation
Source: Nat Commun. 2024 Mar 26;15:2672. doi: 10.1038/s41467-024-47040-x (PMC10965935; doi:10.1038/s41467-024-47040-x)
Supplement: Supplementary file 1 — Supplementary Information [file 41467_2024_47040_MOESM1_ESM.pdf]

Supplementary Information File

Electricity Generation from Carbon Dioxide Adsorption by Spatially Nanoconfined Ion Separation

Zhuyuan Wang[1,2], Ting Hu[2], Mike Tebyetekerwa[1], Xiangkang Zeng[1], Fan Du[2], Yuan Kang[2], Xuefeng Li[1], Hao Zhang[1], Huanting Wang[2], Xiwang Zhang[1, 2, 3]*

[1] UQ Dow Centre for Sustainable Engineering Innovation, School of Chemical Engineering, The University of Queensland, St Lucia, QLD 4072, Australia

[2] Department of Chemical and Biological Engineering, Monash University, Clayton, VIC 3800, Australia

[3] ARC Centre of Excellence for Green Electrochemical Transformation of Carbon Dioxide (GETCO2), Brisbane, Australia

* Email: xiwang.zhang@uq.edu.au

This file contains.

Supplementary Notes 1-7

Supplementary Figures 1-18

Supplementary Tables 1-3

Supplementary Note 1. Ion separation in confined spaces

The key point for energy generation from selective ion transport is to achieve a diffusion rate difference between oppositely charged ions (D_-/D_+ or D_+/D_-) in artificial channels. Ideal ion channels are able to maximize the ion selectivity and transport rate simultaneously to generate useable diffusion currents. Commonly, ion separation in confined spaces relies on electrical effects or size effects[1,2]. In the case of electrical effects, ion diffusion is tuned by the overlapped electrical double layer (EDL) of the charged ion channel when the radius of the channel is narrower than the Debye length of the transported ions. This charge regulation effect (selectivity), however, fades exponentially if the channel size increases to the extent that is comparable to the Debye length [2]. This leads to that narrow channels are preferred to guarantee the ion selectivity in the case of separation regulated solely by charge effects. Nonetheless, narrowing channel for high selectivity is at the expense of scarifying the transport rate of desired ions (Supplementary Figure 1). As for the size effect, this takes advantage of the physical size difference between oppositely charged ions to achieve ion separation. A simple assumption is made that the size of the ion channels locates in between the sizes of ions to be separated. Therefore, achieving high selectivity and permeability lies in widening the size difference between targeted ions.

Supplementary Note 2. Fabrication of bulk g-C_3N_4

A two-step calcination method was employed to prepare bulk graphitic carbon nitride (g-C_3N_4). Initially, 10 grams of urea powder (⩾98%, Sigma-Aldrich) were placed in a 100 mL capacity crucible that was covered and sealed with aluminum foil. The crucible was then heated in a muffle oven under air conditions at a rate of 5°C per minute for 2 hours at a temperature of 550°C. Next, the obtained faint yellow powder (1 g) was ground and filled into a crucible with the same capacity and calcined again under identical conditions. The final product obtained was a light-yellow powder that was stored at room temperature for further experiments.

Supplementary Note 3. *I-V* curve characterization

h-BN-NH_2 nanosheet water solution with its concentration at 1 mg mL^{-1} was chosen for *I-V* curve characterization to elucidate the change of the total movable ions. 15-mL nanosheet solution and DI water was separated by a hydrogel bridge as shown in Supplementary Figure

13. A pair of Ag/AgCl electrodes were immersed in nanosheets solution and DI water respectively. The drift ion current was monitored with a source meter Keithley 2450 with applied voltages from -0.2V to 0.2V. To study the influence of CO_2 adsorption on solution conductivity, the h-BN-NH_2 nanosheet water solution was bubbled with pure CO_2 and N_2 for an hour respectively before the measurement. The increased slope of the *I-V* curve after CO_2 adsorption can be an indicator of an increased number of total movable ions across the hydrogel bridge when an external voltage was applied as the ion-transportation driving force. Besides, due to the highly selective transport of anion over cation by the hydrogel bridge, a negative short circuit current ($I_{sc,}$ 100nA) and a positive open-circuit potential ($V_{OC,}$ 90 mV) were observed from the *I-V* measurements. The obtained I_{sc} and V_{OC} from the solution base *I-V* measurements fit well with the tested value of the hydrogel generator as shown in Fig 2a, b.

Supplementary Note 4. Simulated ion diffusion

The diffusion device was also utilized to simulate the selective ion transport by hydrogel matrix. The concentration of h-BN-NH_2 nanosheets on the left-hand side was set as 5 mg mL^{-1}. The diffusion rate was calculated by monitoring the amount of h-BN-NH_2 nanosheets on the right-hand side by the UV-Vis spectroscopy as a function of diffusion time. The diffusion process of negative ions was simulated by adding sodium bicarbonate salt to replace h-BN-NH_2 solution. The sodium bicarbonate diffusion rate is determined by assessing the conductivity variation within the DI water side concerning diffusion time using a conductivity meter (DZS-708TP, Shanghai Precision Scientific Instrument Co., Ltd). Considering that the agarose channel is significantly larger than both Na^+ and HCO_3^- ions, it is plausible to suggest that the channel lacks selective transport capacity for Na^+ or HCO_3^- ions. Consequently, the transport rate of sodium bicarbonate is inferred to be closely aligned with the transport rate of HCO_3^- ions. Please note that this estimation does not account for the influence of counter ions. In real cases, the difference in diffusion between h-BN-NH_2^+ and HCO_3^- can generate a reverse electrical field, slowing down the following diffusion of the HCO_3^-ions.

Since the measured CO_2 adsorption capacity of h-BN-NH_2 nanosheets is 0.238 mmol g^{-1}, which can be used to estimate the charge density of the h-BN-NH_2 nanosheets after CO_2 adsorption. To ensure a balanced concentration gradient for the simulated positive/negative ions, the concentration of sodium bicarbonate salt was calculated as follows.

$$C_{\mathrm{NaHCO_3}} = C_{\mathrm{BN}} \times \mathrm{M}_{\mathrm{NaHCO_3}} \times 0.238 \times 10^{-3}$$

Where C_{BN} is the concentration of h-BN-NH_2 nanosheets (5 mg mL^{-1}), M_{NaHCO3} is the molar mass of sodium bicarbonate 84.01 g mol^{-1}. The calculated C_{NaHCO3} is at around 0.1 mg mL^{-1}. The diffusion rate D is calculated based on the following equation.

$$D = \frac{C_t V_t}{A} \qquad (1)$$

Where C_t is the measured concentration of sodium bicarbonate or h-BN-NH_2 nanosheet at time *t*, V_t is the volume of the solution in the DI water side at time *t*. *A* is the cross-section area of the hydrogel bridge.

The directly obtained diffusion amount of h-BN-NH_2 nanosheets from UV-Vis is their mass concentration (g mL^{-1}). For better comparison with the diffusion rate of the bicarbonate ion, the mass diffusion rate was multiplied by 0.238×10^{-3} mol g^{-1} as the molar diffusion rate. Note that mol here represents the number of charged units rather than the number of h-BN nanosheets. The obtained diffusion rate of h-BN-NH_2 nanosheets, which is the simulated diffusion rate of positive ions, is shown in Supplementary Table 2. The results show that the diffusion rate of simulated positive ions is six magnitudes slower than the diffusion of simulated negative ions (sodium bicarbonate).

Supplementary Note 5. Reversal potential and transference number

The h-BN-NH_2 (10h milling) water solutions of varying concentrations underwent a 1-hour CO_2 bubbling for testing purposes. These solutions were contained within an H-cell and were separated by a 2% hydrogel bridge, measuring 0.8 cm in diameter and 3 cm in length. In this setup, one compartment received 15 mL of a high concentration infusion (3 mg mL^{-1} and 6 mg mL^{-1}, repetitively), while the other compartment was supplied with 15 mL of a low concentration solution (1 mg mL^{-1} and 2 mg mL^{-1}, creating a threefold concentration gradient). To measure the *I-V* responses across the hydrogel bridge, an electrochemical station was connected to both compartments via salt-bridged Ag/AgCl electrodes at a temperature of 296.15 K. The reversal potential (E_R) was determined at the zero current point on the *I-V* curve. Using the provided potential equation below, we can calculate the membrane transference number (*t*), a pivotal indicator of ion selectivity[3]:

$$t_{-} = \frac{1}{2}\left[\frac{E_R}{\frac{RT}{zF}\ln\left(\frac{a_H c_H}{a_L c_{HL}}\right)} + 1\right] \qquad (2)$$

Where *R* represents the gas constant, *T* is the absolute temperature, z denotes the electrovalence of the counter ion (with z being 1 for positive ions and -1 for negative ions), *C* stands for the ion concentration within the solution, *F* represents the Faraday constant, *H* and *L* indicate high concentration and low concentration respectively, and *t* signifies the transference number. A transference number of $t_- = 1$ indicates an ideal anion-selective transport scenario, where no cation is permitted to pass through.

Supplementary Note 6. Mathematic description of the electricity generation

In a typical CO_2 adsorption process with the presence of water, the generated ion concentration is proportional to the adsorption groups and CO_2 concentration.

$$C\,[\text{h-BN-NH}_3^+\cdot{}^-\text{HCO}_3] = k\cdot C\,[\text{h-BN-NH}_2]\cdot C\,[\text{CO}_2]\cdot C\,[\text{H}_2\text{O}]$$

Where k is the equilibrium constant, *C* [h-BN-NH_2] represents the concentration of the amino groups on h-BN-NH_2 nanosheets. Since water and CO_2 are excessive for adsorption, the ion concentration is primarily determined by the amount of CO_2 adsorption functional groups. It means $C[\text{h-BN-NH}_3^+\cdot{}^-\text{HCO}_3]\ \alpha\ k\cdot C[\text{h-BN-NH}_2]$, indicating that the generated ion concentration is proportional to the density of the amino groups grafted on the boron nitride nanosheets $C[\text{h-BN-NH}_2]$.

It is expected that the concentration gradient is formed across the generator because only the ion-releasing part was exposed to gaseous CO_2. The concentration gradient can force the relocation of the generated ions within the generator to balance the chemical potential difference that is originated from the ion concentration gradient. According to Fick's law, the diffusion rate of the negative and positive ions is $D_1\frac{dC_1}{dx}$ and $D_2\frac{dC_2}{dx}$ respectively, where D_1, D_2 are the diffusion coefficients of bicarbonate ion and h-BN-NH_3^+ ion, respectively, C_1 and C_2 are the ion concentrations of bicarbonate ion and h-BN-NH_3^+ ions, respectively; *x* is the length of the generator, $\frac{dC_1}{dx}$ and $\frac{dC_2}{dx}$ are the concentration gradient of bicarbonate and h-BN-NH_3^+ ions in the generator. Since the negatively charged bicarbonate ions display a much higher diffusion coefficient than that of positively charged h-BN-NH_3^+ ions, it incurs an unbalanced ion distribution of charged ions in the generator during a certain diffusion timeframe and thus an across-generator potential difference (P) and diffusion current (I_{dif}). The potential difference creates an electric filed ($E = \frac{P}{x}$) in the generator that leads to a drift (I_{dri}) current to

counterbalance the ion diffusion[4-6], where P is the potential difference. At an open-circuit stage, the external current $I_{ex} = 0$, which means:

$$I_{ex} = I_{dif+} I_{dri} = - (qD_1A\frac{dC_1}{dx} - qD_2A\frac{dC_2}{dx}) + \sigma E = 0 \qquad (3)$$

Where q, σ, and A are electric quantity of elementary charge, conductivity and cross-section area of the generator, respectively.

According to equation (3),

$$E = \frac{qAD_1}{\sigma}\frac{dC_1}{dx} - \frac{qAD_2}{\sigma}\frac{dC_2}{dx} \qquad (4)$$

Therefore, the induced open-circuit voltage P can be calculated as:

$$P = \int(\frac{qAD_1}{\sigma}\frac{dC_1}{dx} - \frac{qAD_2}{\sigma}\frac{dC_2}{dx}) \qquad (5)$$

In a short period of diffusion time, we assume that $\frac{dC_1}{dx} \approx \frac{dC_2}{dx} \approx \frac{dC_0}{dx}$, in addition, we assume that the diffusion coefficient and ion conductivity are consistent in the whole generator, meaning that $\frac{qD_1}{\sigma}$ and $\frac{qD_2}{\sigma}$ are not functions of x, and thus P can be simplified as:

$$P = \frac{qA(D_1 - D_2)}{\sigma}\int\frac{dC_0}{dx} \qquad (6)$$

According to equation (6), the CO_2-induced voltage is fundamentally a result of the diffusion coefficient gap between negative and positive ions, and the potential difference is proportional to the diffusion coefficient gap and the total amount of h-BN-NH_2 in the generator. The obtained knowledge agrees well with experimental results. From the perspective of selective ion transportation, the optimization of P lies in increasing (D_1-D_2) and $\frac{dC_0}{dx}$ in equation (6). Increasing these two factors in equation (6) corresponds to enhancing the diffusion coefficient gap between negative and positive ions and raising the maximum CO_2 adsorption capability of the adsorbent, respectively.

Supplementary Note 7. Calculation of the energy harvesting efficiency

From the perspective of energy conversion, the input energy was thought to be the chemical potential difference of CO_2 before and after adsorption. This chemical potential variation is related to the phase transformation of CO_2 from its gaseous phase into an ordered adsorption

phase. The adsorption process is thought to be an isothermal and isobaric process, and can be calculated as[5,7]:

$$\mu_i = (\frac{\partial G}{\partial n_i})_{T,P} \qquad (7)$$

$$\mu_i = \mu_i^\theta + RTlna_i \quad (8)$$

In this case, the energy input can be estimated as[5,6,8]:

$$\Delta G_{in} = \mu_g - \mu_a \approx RTln\frac{C_0}{C_0 - \Delta C} \qquad (9)$$

Where ΔG_{in}, μ_g, μ_a, *R*, *T*, C_0, and ΔC represent the Gibbs free energy variation, the chemical potential of gaseous CO_2, the chemical potential of the adsorbed CO_2, the ideal gas constant, the testing temperature (20 °C), the CO_2 concentration in the feed gas, and the variation of CO_2 concentration, respectively. For a simple estimation, the concentration of CO_2 in the feed gas was considered as that of pure CO_2 that is ~1.82 kg m^{-3} (C_0), and the variation was thought to be the concentration the theoretical CO_2 adsorption capacity in the generator that is 10.47 g m^{-3} (ΔC). As a result, an appropriately estimated ΔG_{in}, which is considered as the input energy, was at ~14.06 J mol^{-1} at 293.15 K.

The maximum output energy that the generator can achieve is measured directly by connecting the generator with different resistors with their resistances spanning 0.1 to 10 MΩ. The generated electricity (*W*) can be calculated according to the current curve as a function of time monitored by the source meter:

$$\mathrm{W} = \int RI^2(t)\, d(t) \qquad (10)$$

Where R is the external resistance.

The total generated energy normalized by the theoretical CO_2 adsorption capacity (E_M) is calculated as equation 11.

$$E_M = \frac{W}{C_{Adsorption}} \qquad (11)$$

Where $C_{\mathrm{Adsorption}}$ is the maximum CO_2 adsorption capacity of the total nanosheets within the hydrogel.

The volumetric power density (P_V) is obtained by equation 12.

$$P_V = \frac{I_M^2 * R}{V} \qquad (12)$$

Where I_M stands for the peak current. V is the total volume of the hydrogel.

The generated energy (E_M) reached its peak at 23.9 μW·h mol^{-1} when connected to a 0.5 MΩ resistor, equivalent to 0.086 J mol^{-1}. The total energy input from the CO_2 adsorption calculated based on equation 9 is at 14.3 J mol^{-1}. This indicates an estimated energy conversion efficiency of approximately 0.6% at 293.15 K, comparable to the efficiency of bilayer polyelectrolyte films used in moisture power generation (~1%)[5]. The volumetric power density stands at 0.0027 μW cm^{-3}. To enhance this relatively insufficient volumetric power density, increasing the device's CO_2 adsorption capacity and adopting a more compact design are recommended as future research directions.

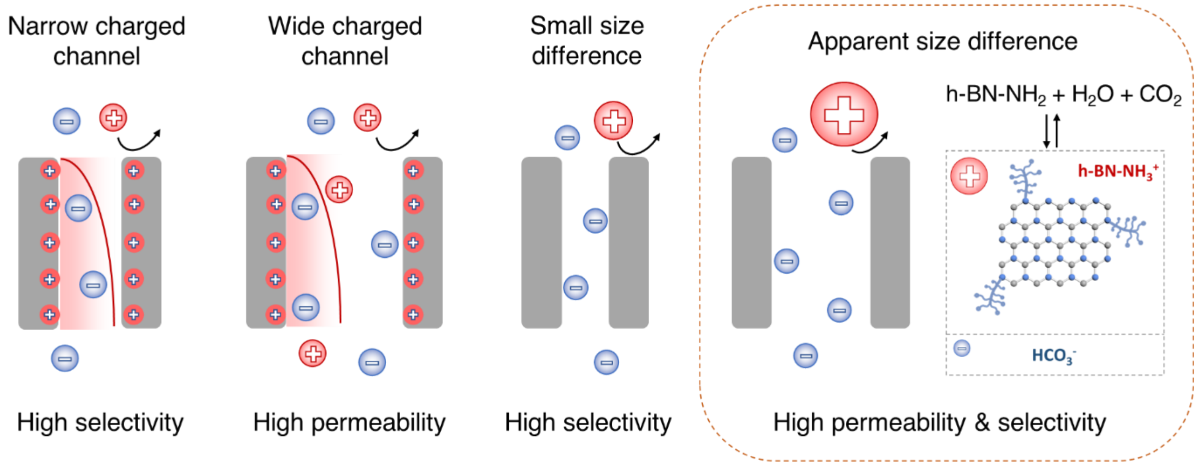

Supplementary Figure 1. Schematic of designing high-speed selective ion channels to achieve directional ion diffusion. The key to realizing high permeability without sacrificing selectivity lies in enlarging the size difference between ions so that a wide channel can be applied.

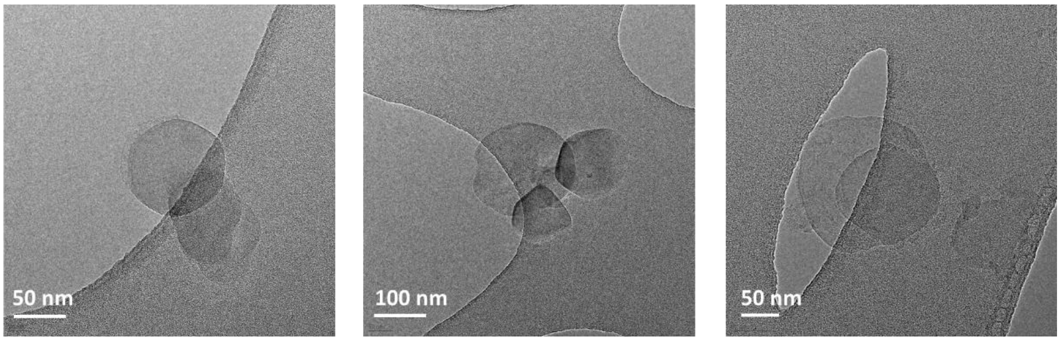

Supplementary Figure 2. TEM images of the exfoliated h-BN nanosheets. h-BN:PEI=1:2, rotation speed 500 rpm, milling time: 10h

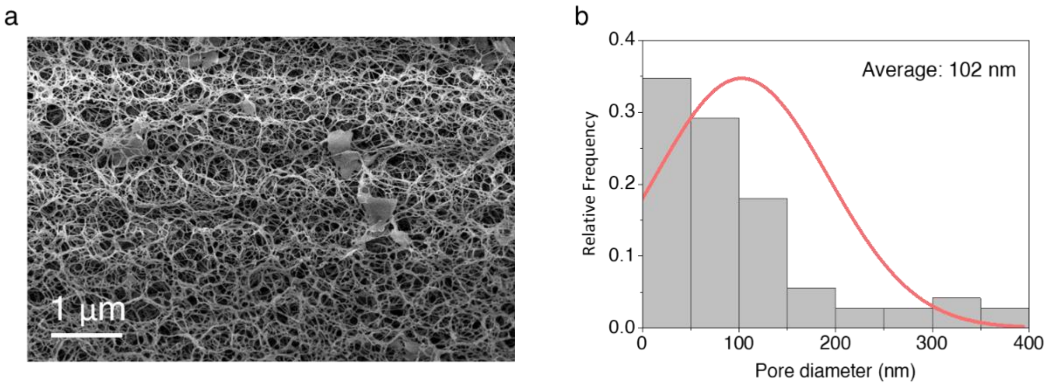

Supplementary Figure 3. Structure of the NAH. a, SEM image of the cross-section structure of the NAH composite. **b,** Diameter distribution of the observed pores analyzed by image J software.

Supplementary Table 1. CHNS Elemental analysis of bulk h-BN, PEI and exfoliated h-BN nanosheets obtained from different milling time.

Sample	C (wt%)	N (wt%)	H (wt%)	PEI grafting ratio (wt%)
PEI	52.432	31.951	11.681	-
Bulk h-BN	0.248	23.124	0.145	-
h-BN-1h	1.012	23.989	0.582	1.46
h-BN-5h	3.383	18.984	0.645	5.98
h-BN-10h	4.399	15.999	0.84	7.92
h-BN-15h	4.943	14.284	0.96	8.95

Note: The weight ratio of grafted PEI is calculated based on the carbon content according to function (13)

$$W_g = (C_{(\text{h-BN nanosheets})} - C_{(\text{bulk h-BN})})/C_{(\text{PEI})} \times 100\% \quad (13)$$

Where W_g is the PEI grafting ratio, $C_{(\text{h-BN nanosheets})}$, $C_{(\text{bulk h-BN})}$, $C_{(\text{PEI})}$ are the measured carbon weight ratios of the power sample of h-BN nanosheets, bulk h-BN, and liquid polymer PEI, respectively.

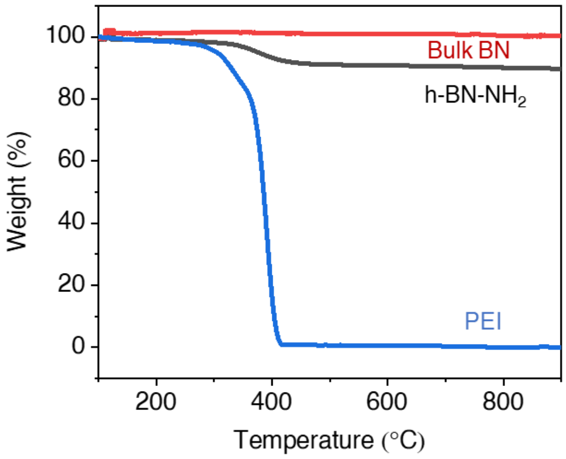

Supplementary Figure 4. TGA analysis on the bulk BN, h-BN-NH_2 nanosheets and PEI liquid. According to the result, pure PEI can totally decompose by 400 °C. The weight loss of h-BN-NH_2 relative to bulk h-BN is at 9.5 wt%, which is thought to be the weight ratio of the functionalities of the obtained nanosheets. This weight ratio is slightly higher than the grafting ratio calculated from the results of the CHNS elemental analysis. Considering the active boron can easily hydrolysis into boric acid during rinsing process as confirmed by our XPS survey scans, this functionalization of boron atoms can also contribute to the weight loss during the TGA process and thus being counted as the grafting ratio. Note that the h-BN-NH_2 nanosheets for TGA analysis come from 10-h milling exfoliation.

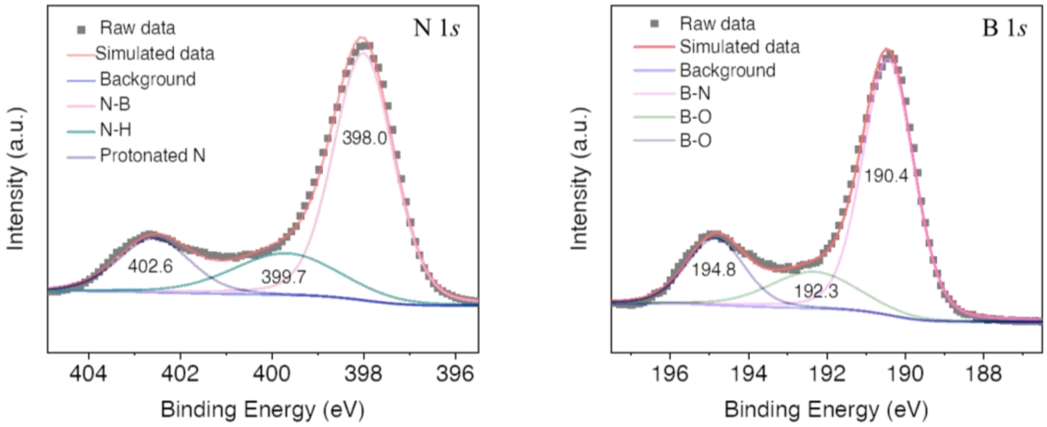

Supplementary Figure 5. XPS spectra of the B 1*s* and N 1*s* of the obtained h-BN-NH_2 nanosheets. The characteristic N-H peaks in N 1*s* XPS spectrum indicate the successful functionalization of PEI molecules on boron nitride nanosheets. This functionalization process mainly occurs along the edge of the freshly broken boron nitride nanosheets with active B or N atoms driven by mechanical energy generated by the ball mill[10,11]. The B-O peak in B 1*s* spectrum could be caused by the hydrolysis of PEI functionalized boron nitride in water during the rinsing process, which leads to the formation of boron acid groups along the edge of the obtained nanosheets[12].

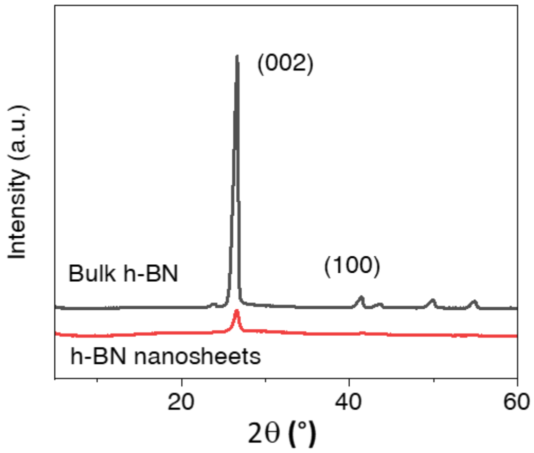

Supplementary Figure 6. Powder XRD patterns of the bulk h-BN and PEI exfoliated h-BN nanosheets. There is an identifiable peak (002) at 26.2° from h-BN-NH_2 nanosheets but with its intensity gets weaker compared to bulk h-BN. Since peak (002) arrives from paralleled stacked boron nitride interlayers, it suggests the significantly decreased layer numbers after 10-h milling exfoliation.

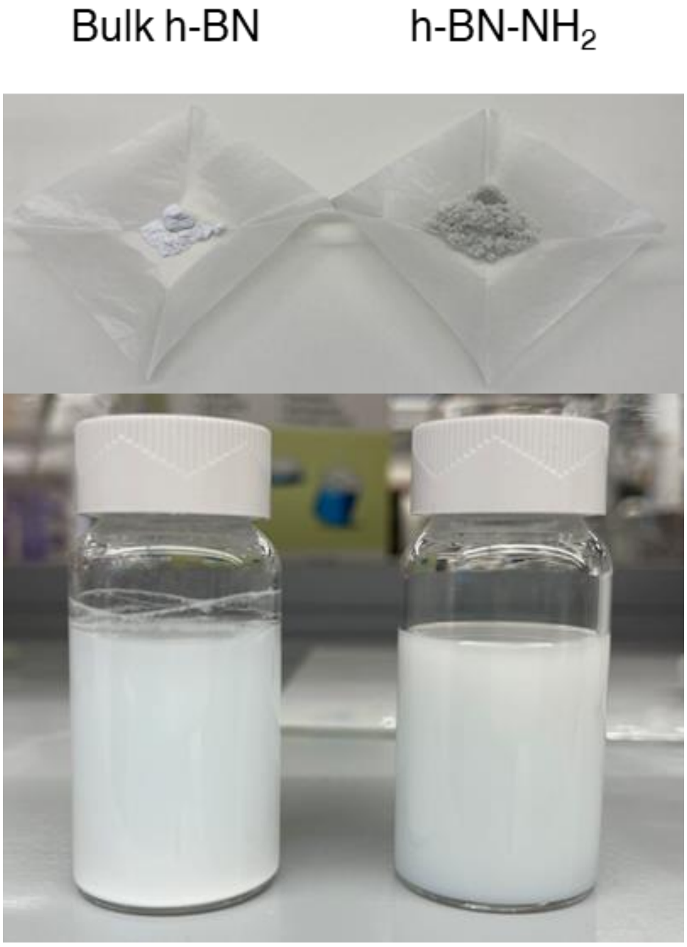

Supplementary Figure 7. Photographs of powder products and water dispersions at a concentration of 5 mg mL^{-1} of bulk h-BN powder and h-BN-NH$_2$ nanosheets. The photos show that powder of h-BN-NH$_2$ nanosheets exhibits fluffy texture compared to bulk h-BN. The PEI functionalized nanosheets can be evenly dispersed in water at a concentration up to 5 mg mL^{-1} while bulk h-BN precipitated in minutes, displaying white colored particle accumulated at the bottom of the dispersion solution.

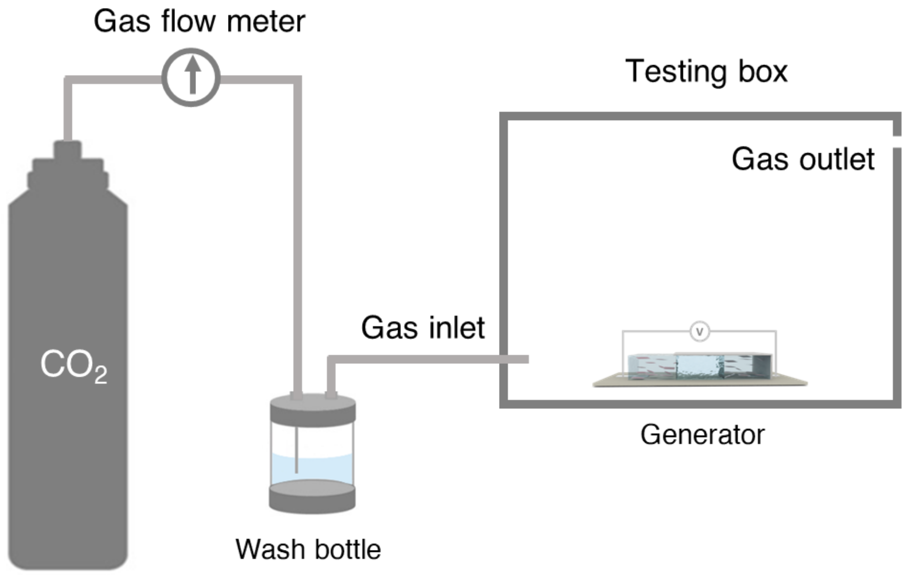

Supplementary Figure 8. Schematic of the homemade setup for testing CO_2-induced electricity generation. The CO_2 gas cylinder was replaced with other types of gas cylinders for the controlled experiments. The CO_2 feeding speed and time were regulated by the gas flow meter.

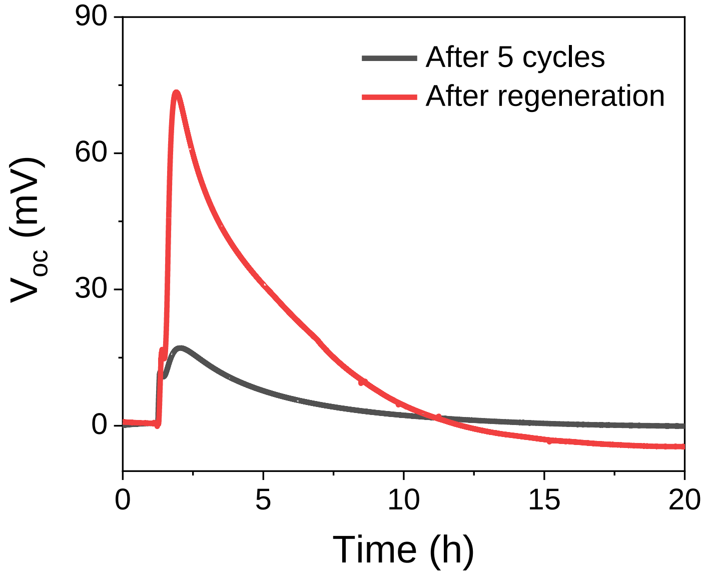

Supplementary Figure 9. pH swing for the regeneration of the generator. The V_{OC} testing after 5-cycle of short circuit discharging and pH swing regeneration, respectively. A buffer solution with a pH value of 10 was employed for the regeneration process. It's crucial to note that the resulting $CaCO_3$ was retained within the generator without undergoing any specific treatment, as the electrical signals can be fully restored.

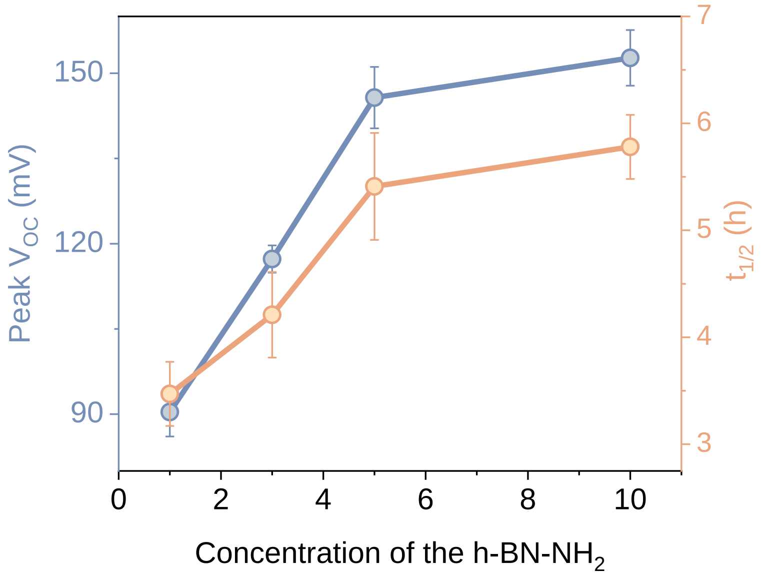

Supplementary Figure 10. The impact of h-BN-NH$_2$ concentration in the NAH hydrogel on power generation performance. The results demonstrated a substantial enhancement in power generation performance with an increase in concentration from 1 mg mL^{-1} to 5 mg mL^{-1}, yielding an approximate 61% improvement in peak V_{OC}. This aligns with the ionization formula provided in Supplementary Note S5, suggesting that the quantity of released ions is directly proportional to the amount of h-BN-NH$_2$ nanosheets. However, upon further increasing the concentration to 10 mg mL^{-1}, precipitation was observed in the precursor h-BN-NH$_2$ nanosheet solution, and the generated power showed only marginal improvement, amounting to less than 5%. To enhance the performance, breakthroughs in material modifications are encouraged. Strategies such as increasing the PEI grafting ratio of the nanosheets or reducing the size of the nanosheets by using 2D quantum dots could potentially improve CO_2 adsorption capacity and water dispersion solubility, thereby enhancing overall performance. Error bars are standard deviations from three tests.

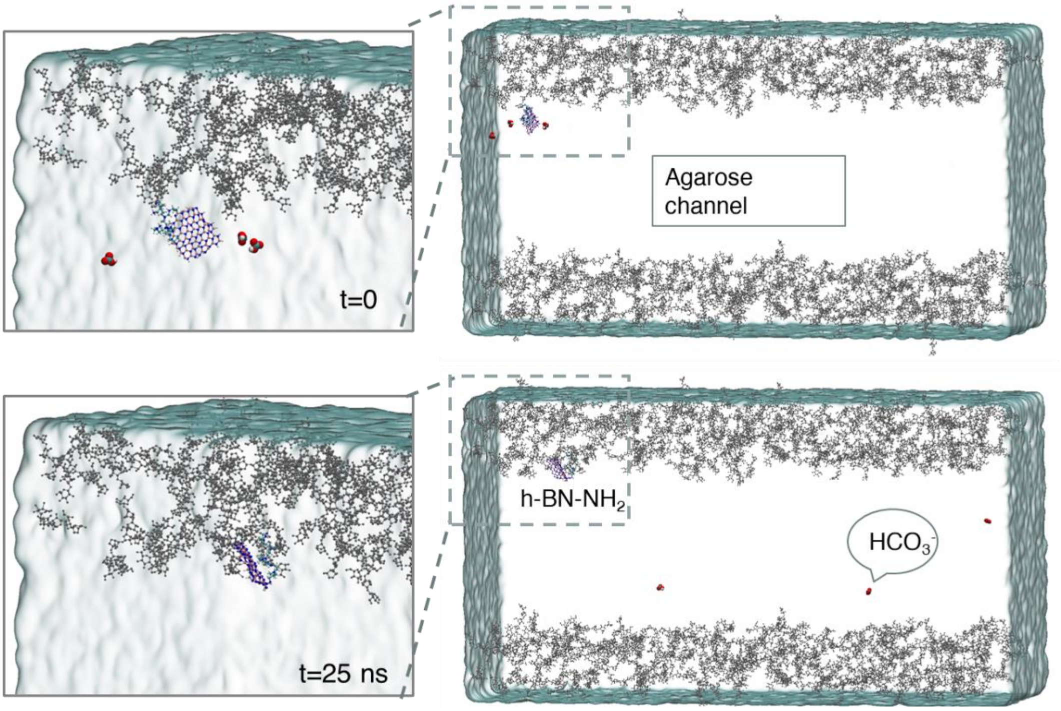

Supplementary Figure 11. MD simulation model of the agarose channel for selective transport of h-BN-NH_3^+ and bicarbonate ions. The transport position at 0 ns and 25 ns of HCO_3^- ions and h-BN-NH_2 nanosheet through the agarose channel. Then HCO_3^- ions and h-BN-NH_3^+ nanosheet are initially placed at the left side of the agarose channel at 0 ns. After 25 ns simulation, the HCO_3^- ions permeated through the channel while the nanosheet attached to the channel entry.

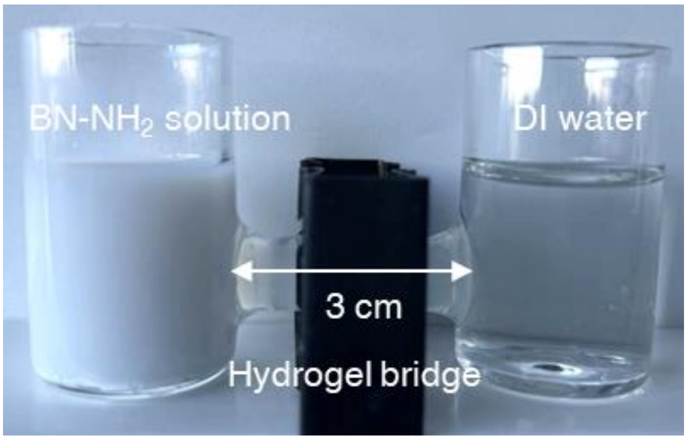

Supplementary Figure 12. Photograph of the homemade diffusion device.

Supplementary Table 2. Permeated amount of simulated positive (h-BN-NH_2 nanosheets) and negative (sodium bicarbonate) ions as a function of diffusion time.

Time (h)	Permeated negative ions (mol m^{-2})	Permeated positive ions (mol m^{-2})
3	2.13	-
8	2.49	-
12	3.27	8.32×10^{-6}
24	4.82	2.1×10^{-6}
48	8.35	3.77×10^{-6}
96	15.78	8.79×10^{-6}

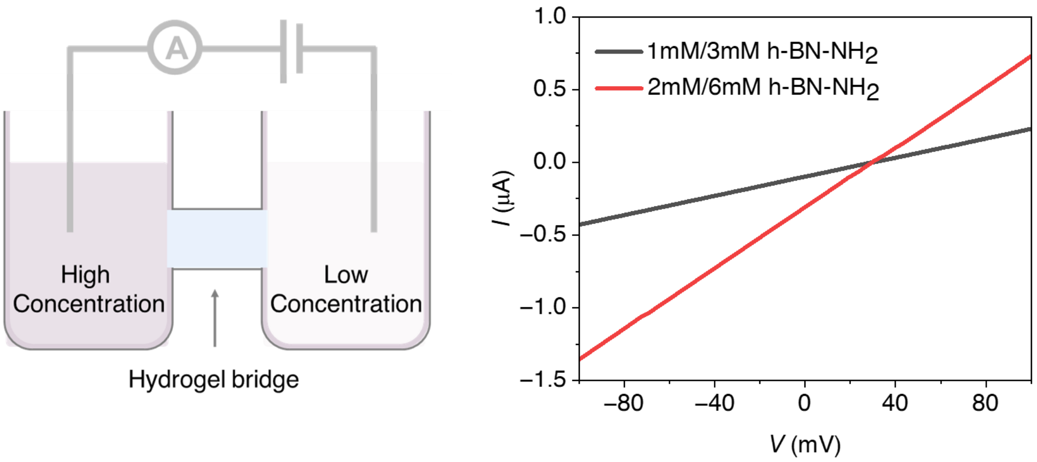

Supplementary Figure 13. *I-V* curve of the nanosheet solution recorded under a 3-fold concentration gradient. The experimental details are outlined in Supplementary Note S5. Using the measured reversal potential, we derived a transference number of anions at 1±0.05. This result corresponds to a near-perfect anion/cation selectivity, which aligns consistently with our observations from diffusion tests.

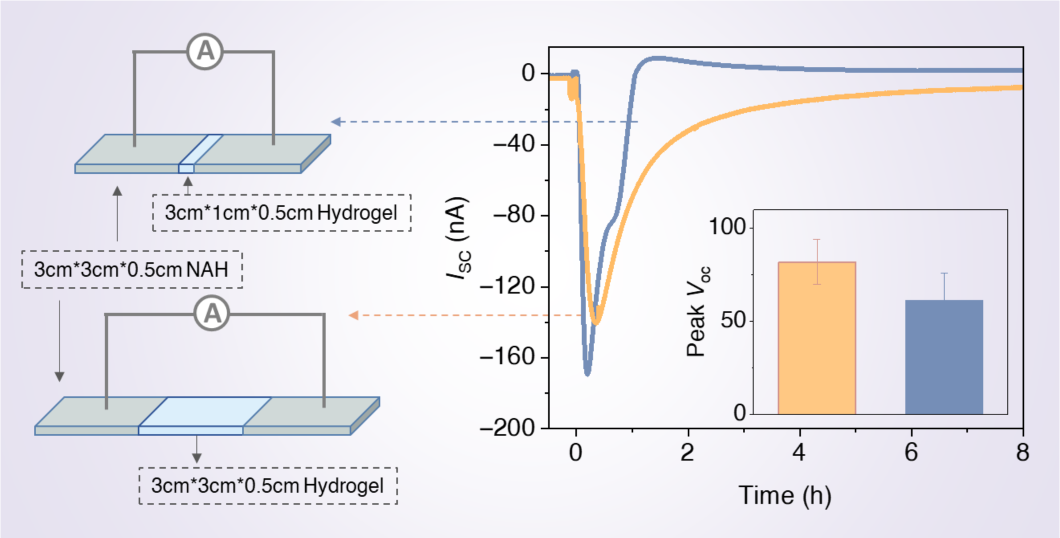

Supplementary Figure 14. Power generation in terms of I_{SC} (right) and V_{OC} (inset) by devices featuring different lengths of the ion diffusion path. The results demonstrate that a device with a shorter diffusion length exhibits a faster charging and discharging process compared to one with a longer diffusion path. The short circuit current (I_{SC}) suggests a higher peak ion flux achieved by shortening the diffusion path. However, despite these variations, their peak power density ($P=I_{max}*V_{max}$) remains at similar level. This observation implies that power density is more likely determined by the CO_2 adsorption capacity of the NAH hydrogel on the ion-releasing part rather than the pure hydrogel in the middle. Error bars are standard deviations from three tests.

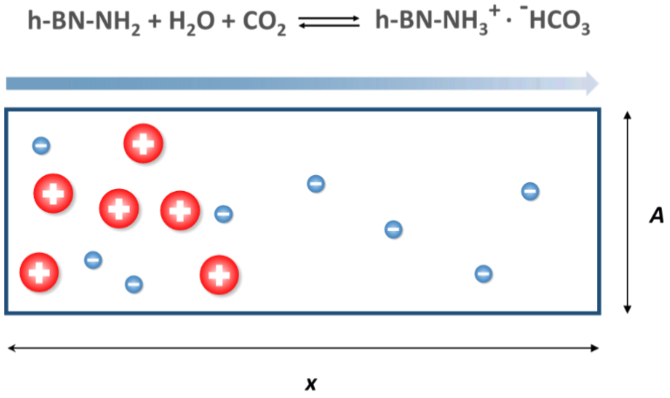

Supplementary Figure 15. Schematic of the selective ion-diffusion-induced electricity generation process within the agarose matrix.

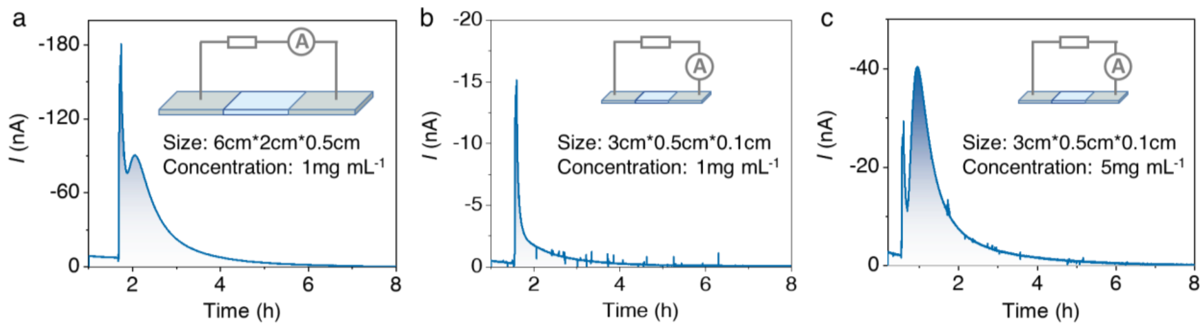

Supplementary Figure 16. Power generation outcomes resulting from (b) a more compact design and (c) higher nanosheet loading compared with the prototype generator (a). These alterations were implemented to explore the possibility of increasing the power density of the generator. In the case of the more compact design, the generator was modified to achieve a condensed configuration aimed at improving ion flux efficiency. Simultaneously, the nanosheet concentration was heightened to elevate the concentration gradient within the system, thus enhancing the ion flux. To assess the impact of these modifications on power density, the generator was connected to an external resistor of 0.5 MΩ, and the resulting current curve was meticulously monitored. It's crucial to note that throughout these experimental modifications aimed at enhancing the power density, all other experimental conditions remained consistent. The collected data from these experiments were utilized to calculate the power density, and the detailed results are provided in Supplementary Table 3.

Supplementary Table 3. The power density of the devices calculated based on Supplementary Figure 16

Devices	Configuration	Nanosheet concentration	Volumetric peak power density (μW cm^{-3})
a	6cm*2cm*0.5cm	1 mg mL^{-1}	0.0027
b	3cm*0.5cm*0.1cm	1 mg mL^{-1}	0.0092
c	3cm*0.5cm*0.1cm	5 mg mL^{-1}	0.024

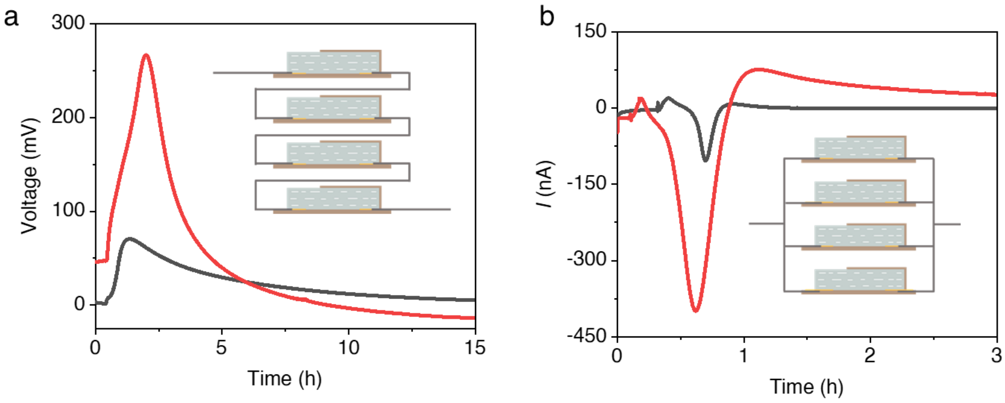

Supplementary Figure 17. Integration of four pieces of the whole NAH composite film through series (a) and parallel (b) connection and their CO_2-induced power generation. Black line: a single piece. Red line: four pieces.

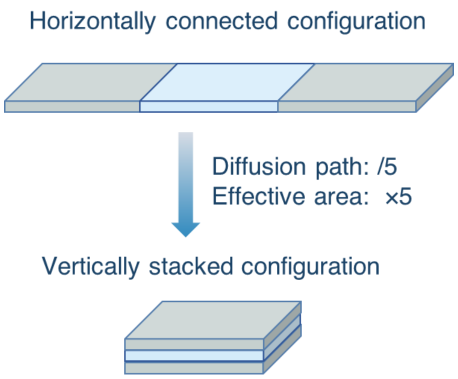

Supplementary Figure 18. Schematic comparison on the length of the diffusion path and the effective diffusion area of the two different configurations.

Supplementary References

1 Kang, Y., Xia, Y., Wang, H. & Zhang, X. 2D laminar membranes for selective water and ion transport. *Adv. Funct. Mater.* **29**, 1902014 (2019).

2 Shen, J., Liu, G., Han, Y. & Jin, W. Artificial channels for confined mass transport at the sub-nanometre scale. *Nat. Rev. Mater.* ***6***, 294-312 (2021).

3 Tunuguntla, R. H. *et al.* Enhanced water permeability and tunable ion selectivity in subnanometer carbon nanotube porins. *Science* **357**, 792-796 (2017).

4 Liu, X. *et al.* Power generation from ambient humidity using protein nanowires. *Nature* **578**, 550-554 (2020).

5 Wang, H. *et al.* Bilayer of polyelectrolyte films for spontaneous power generation in air up to an integrated 1,000 V output. *Nat. Nanotechnol.* **16**, 811-819 (2021).

6 Zhao, F., Liang, Y., Cheng, H., Jiang, L. & Qu, L. Highly efficient moisture-enabled electricity generation from graphene oxide frameworks. *Energy Environ. Sci.* **9**, 912-916 (2016).

7 Brachman, M. K. Fermi level, chemical potential, and Gibbs free energy. *J. Chem. Phys.* **22**, 1152-1152 (1954).

8 Zhao, F., Cheng, H., Zhang, Z., Jiang, L. & Qu, L. Direct power generation from a graphene oxide film under moisture. *Adv. Mater.* **27**, 4351-4357 (2015).

9 Fontela, M., Velo, A., Brown, P. & Pérez, F. in *Marine Analytical Chemistry* 1-37 (Springer, 2022).

10 Jeon, I. Y., Bae, S. Y., Seo, J. M. & Baek, J. B. Scalable production of edge‐functionalized graphene nanoplatelets via mechanochemical ball‐milling. *Adv. Funct. Mater.* **25**, 6961-6975 (2015).

11 Lei, W. *et al.* Boron nitride colloidal solutions, ultralight aerogels and freestanding membranes through one-step exfoliation and functionalization. *Nat. Commun.* **6**, 1-8 (2015).

12 Chen, S. *et al.* Simultaneous production and functionalization of boron nitride nanosheets by sugar‐assisted mechanochemical exfoliation. *Adv. Mater.* **31**, 1804810 (2019).
