# Supplementary material for: Electricity generation from carbon dioxide adsorption by spatially nanoconfined ion separation
Source: Nat Commun. 2024 Mar 26;15:2672. doi: 10.1038/s41467-024-47040-x (PMC10965935; doi:10.1038/s41467-024-47040-x)
Supplement: Supplementary file 3 — Description of Additional Supplementary Files [file 41467_2024_47040_MOESM3_ESM.pdf]

Description of Additional Supplementary Files

File Name: Supplementary Movie 1
Description: The movie demonstrates that electricity harvested from CO2 adsorption reached 5V through the series and parallel connection of multiple generators, which is then used to power a light-emitting diode for several cycles.
